# Remnant cholesterol/high-density lipoprotein cholesterol ratio is a new powerful tool for identifying non-alcoholic fatty liver disease

**DOI:** 10.1186/s12876-022-02216-x

**Published:** 2022-03-24

**Authors:** Yang Zou, Chong Hu, Maobin Kuang, Yuliang Chai

**Affiliations:** 1 Jiangxi Cardiovascular Research Institute, Jiangxi Provincial People’s Hospital, The First Affiliated Hospital of Nanchang Medical College, Nanchang, 330006 China; 2 Gastroenterology Department, Jiangxi Provincial People’s Hospital, The First Affiliated Hospital of Nanchang Medical College, Nanchang, 330006 China; 3 Cardiology Department, Jiangxi Provincial People’s Hospital, The First Affiliated Hospital of Nanchang Medical College, Nanchang, 330006 China

**Keywords:** Remnant cholesterol/high-density lipoprotein cholesterol ratio, Remnant cholesterol, High-density lipoprotein cholesterol, Non-alcoholic fatty liver disease, RC/HDL-C ratio

## Abstract

**Background:**

Remnant cholesterol/high-density lipoprotein cholesterol (RC/HDL-C) ratio has been shown to be a good predictor of metabolic disease risk, but no studies have further investigated the role of RC/HDL-C ratio in non-alcoholic fatty liver disease (NAFLD) patients.

**Methods:**

The participants were 14,251 adults who underwent a physical examination, all of whom underwent abdominal ultrasonography to determine whether they had NAFLD. Receiver operating characteristic curve analysis and multivariate logistic regression models were used to assess the association between the RC/HDL-C ratio and the risk of NAFLD.

**Results:**

Multivariate logistic regression analysis showed that after fully adjusting the confounding factors, the higher RC/HDL-C ratio was independently positively correlated with the risk of NAFLD. Interaction tests suggested that the effect of RC/HDL-C ratio on NAFLD was significantly affected by sex. Additionally, receiver operating characteristic curve analysis showed that the area under the curve of RC/HDL-C ratio for identifying NAFLD was 0.82, which was significantly higher than that of other conventional lipid parameters.

**Conclusions:**

This study indicates for the first time that the higher RC/HDL-C ratio in the general population may be closely related to the increased risk of NAFLD.

**Supplementary Information:**

The online version contains supplementary material available at 10.1186/s12876-022-02216-x.

## Background

Non-alcoholic fatty liver disease (NAFLD) seems to have become the most common chronic disease in the world. According to a recent epidemiological survey of more than 8 million people in 22 countries by Younossi et al., the current prevalence of NAFLD is 25.24% in the world and 27.37% in Asia [1]. With the increasing prevalence of obesity and diabetes worldwide, the prevalence of NAFLD continues to go up and has become a global public health problem [1, 2]. NAFLD contains a series of liver histological changes ranging from mild hepatic steatosis to severe necrotizing inflammation. Without any interventions, it will eventually develop into liver cirrhosis or even liver cancer, leading to serious adverse consequences [3–5]. Additionally, many clinical studies have found that NAFLD also causes many adverse effects on other organs and systems outside the liver [6–8]. The widespread adverse consequences caused by NAFLD in and out of the liver have further increased the global burden of chronic diseases. Therefore, both medical institutions and the general public should pay attention to the preventive screening and management of NAFLD.

Atherogenic dyslipidemia is closely related to NAFLD, in which characteristic changes of triglycerides (TG) and high-density lipoprotein cholesterol (HDL-C) are common in NAFLD patients [9, 10]. Therefore, monitoring atherosclerotic lipids is an important way to assess the risk of NAFLD [9, 11]. Remnant cholesterol (RC) is an unconventional lipid that has been widely studied in recent years. It is a kind of lipoproteins rich in TG, that is, intermediate-density lipoprotein and very-low-density lipoprotein in fasting state, and in the nonfasting state also chylomicron remnants [12]. It is a key lipoprotein of atherosclerosis [13, 14]. Some previous clinical studies have also confirmed that RC is a major factor mediating the residual risk of major cardiovascular events and is independently related to the progression of atherosclerosis [14–16]. In recent years, a growing number of studies have found that high levels of RC significantly increased the risk of diabetes complications, hypertension and NAFLD; additionally, high levels of RC can also be used to predict cardio-cerebrovascular events in patients with NAFLD [17–20]. These pieces of evidence suggest that RC may be a good parameter for assessing the risk of metabolism-related disease. Recently, some scholars have pointed out that the parameter remnant cholesterol/high-density lipoprotein cholesterol ratio (RC/HDL-C ratio) after the combination of RC and HDL-C is a valuable independent predictor of myocardial injury in diabetic patients after receiving PCI; in addition, the RC/HDL-C ratio can also be used to evaluate intracranial atherosclerotic [21, 22]. However, there are no epidemiological studies to investigate the relationship between RC/HDL-C ratio and NAFLD, and it remains unclear whether the RC/HDL-C ratio is a risk factor for NAFLD. Here, in order to solve these problems, this study retrospectively analyzed the population data of NAGALA (NAfld in Gifu Area, Longitudinal Analysis) cohort to examine the relationship between RC/HDL-C ratio and NAFLD.

## Methods

### Study data and participants

This study was a secondary analysis of the NAGALA cohort data set to examine the relationship between the RC/HDL-C ratio and NAFLD. The NAGALA study is a cohort study based on general adults that have been ongoing since 1994 to assess risk factors for common chronic diseases, including NAFLD, in the general population. Details of the study design have been published elsewhere [23]. Available research data were uploaded to the Dryad database by Hamaguchi et al. According to the Dryad data usage service, different researchers can use the data set for in-depth analysis according to different research hypotheses, and need to indicate the source of the data [24].

This study extracted the available data from the NAGALA cohort from 2004 to 2015, and established new exclusion criteria based on the research hypothesis, including the following: (1) During the baseline interview, men drank more than 210 g per week or women drank more than 140 g per week (1952) [25]; (2) Participants who were still taking oral medication at the time of the baseline visit (2321); (3) Diagnosis of impaired fasting glucose or diabetes or alcoholic hepatitis or viral hepatitis or autoimmune hepatitis or liver disease due to other causes were made at the baseline visit (1547); (4) Participants with missing baseline information (873). Informed consent of participants had been obtained in previous studies [23]. In addition, as the previous study had been authorized by the ethics committee of Murakami Memorial Hospital, the Institutional Review Board (IRB) of Jiangxi Provincial People’s Hospital exempted the application for duplicate ethical authorization (IRB Number: 2021-066).

### Clinical characteristics

As mentioned earlier, data on the demographic characteristics and lifestyle of study participants were recorded by trained medical staff using a standard questionnaire [23]. Anthropometric data collected included height, weight, waist circumference and blood pressure; lifestyle data included a habit of exercise, smoking and drinking status, history of chronic diseases and history of medication. Among them, the habit of exercise was evaluated according to whether they participated in exercise regularly or not, and the frequency was at least once a week. Smoking status and drinking status were classified according to past history asked during the baseline interview, in which smoking status was divided into three groups: non-smoking, past smoking and current smoking, and drinking status was divided into non-drinking or small, light and moderate groups.

### Biochemical parameters

All venous blood samples were collected after fasting overnight, and the biochemical parameters such as aspartate aminotransferase (AST), HDL-C, total cholesterol (TC), hemoglobin A1c (HbA1c), gamma-glutamyl transferase (GGT), TG, fasting plasma glucose (FPG) and alanine aminotransferase (ALT) were determined by standard experimental method.

### Definition and calculation

The study population underwent abdominal ultrasound to assess NAFLD. Liver sonograms were first acquired by an experienced technician using the AlokassD-650CL ultrasound system, and then evaluated by a gastroenterologist without knowledge of the participants’ other examination information. The main features of NAFLD under ultrasound were determined and scored according to the four criteria of deep elevation, hepatorenal echo contrast, liver brightness and vascular blurring. Participants with a final score greater than 2 were diagnosed with NAFLD [26].

RC was calculated as non-HDL-C – low-density lipoprotein cholesterol (LDL-C) [27], where non-HDL-C = TC – HDL-C and LDL-C (mg/ dL) = non-HDL-C x 90% – TG x 10% [28].

RC/HDL-C ratio was calculated as RC divided by HDL-C [21].

### Statistical analysis

In this study, descriptive statistics were firstly carried out for the quartile grouping of the RC/HDL-C ratio. The descriptive data of the study population were expressed as the percentage of categorical variables and the mean (standard deviation) or median (interquartile range) of continuous variables. Comparisons between groups were performed using one-way analysis of variance or Kruskal-Wallis test or Chi-square test. Then, according to the Strengthening the Reporting of Observational Studies in Epidemiology guidelines, the OR (odds ratio) and 95% CI (confidence interval) of the ratio of RC/HDL-C to the risk of NAFLD in different multivariate logical regression models were reported [29]. Among which model I was regarded as a fine-tuning model, and sex and age were regarded as confounding factors. Model II takes non-collinear variables with an impact of > 10% on the risk of NAFLD associated with the RC/HDL-C ratio as confounders [30, 31]. Based on model II, model III further treated the significant covariables in the univariate analysis as confounding factors. Model IV was regarded as a fully adjusted model, in which all non-collinear covariables were taken as confounding factors (Additional file 1: Table S1) [30]. Sensitivity analysis was performed by limiting the RC/HDL-C ratio quartiles to continuous variables in four models. Additionally, we created stratified models to assess the NAFLD risk of RC/HDL-C ratios in people of different sexes, ages, BMI and habits of exercise. In order to evaluate the potential interaction, the likelihood ratio test was also used to compare the differences of subgroups in different models. Finally, to further evaluate the accuracy of the RC/HDL-C ratio in identifying NAFLD risk, we also used the receiver operating characteristic (ROC) curve to assess the area under the curve (AUC) of the RC/HDL-C ratio and other lipid parameters, and compared the AUC of RC/HDL-C ratio with that of other lipid parameters by DeLong test [32]. All statistical analyses were performed using R language version 3.4.3 and Empower (R) version 2.0. Double-tail *P* < 0.05 was considered to be statistically significant.

## Results

### Characteristics of study participants

The observational study involved 14,251 participants. The average age of the study population was 43 years old, and 6840 (48%) were women. According to the results of abdominal ultrasound, 2507 (17.59%) participants were diagnosed with NAFLD. Table 1 describes the clinical and biochemical characteristics of the study population according to the quartiles of RC/HDL-C ratios. We noted significant differences in all baseline information across the quartile groups of RC/HDL-C ratios. The main results were summarized below: (1) In the group with a higher RC/HDL-C ratio, the number of men was about 5 times higher than that of women (83.15% vs. 16.85%). This suggests that the RC/HDL-C ratio may be sex-dependent, and the NAFLD risk associated with the RC/HDL-C ratio may have a better distinction between the sexes. In addition, the group with a higher RC/HDL-C ratio was significantly older. (2) Anthropometric parameters were significantly higher in the higher RC/HDL-C ratio group. (3) Biochemical parameters except for HDL-C, other lipid parameters, glucose metabolism parameters and liver enzymology parameters were significantly higher in the high RC/HDL-C ratio groups. It should be noted that although liver enzymology indexes ALT, AST and GGT were gradually increased across the RC/HDL-C ratio quartiles elevated, they were all at normal levels in all groups, suggesting that most of the patients with NAFLD in this study may still be in the early stage. (4) In the group with a higher RC/HDL-C ratio, there were fewer people maintained exercise habits, and more people smoking and drinking alcohol. (5) The prevalence of NAFLD between the RC/HDL-C ratio quartile was gradually increased (1.94% vs. 6.29% vs. 18.48%, 43.58%).

**Table 1:** Baseline characteristics of four groups

	RC/HDL-C ratio quartile				
	Q1 (0.07–0.26)	Q2 (0.26–0.37)	Q3 (0.37–0.55)	Q4 (0.55–4.39)	*P*-value
Sex					< 0.001
Women	2785 (78.21%)	2111 (59.30%)	1344 (37.75%)	600 (16.85%)	
Men	776 (21.79%)	1449 (40.70%)	2216 (62.25%)	2961 (83.15%)	
Age, years	40.00 (35.00–46.00)	42.00 (36.00–49.00)	44.00 (38.00–51.00)	45.00 (39.00–52.00)	< 0.001
Weight, kg	52.70 (8.11)	57.08 (9.47)	62.16 (10.55)	69.10 (11.13)	< 0.001
Height, cm	161.70 (7.53)	163.50 (8.46)	165.72 (8.56)	168.25 (7.88)	< 0.001
BMI, kg/m^2^	20.09 (2.21)	21.27 (2.56)	22.55 (2.91)	24.34 (3.08)	< 0.001
WC, cm	69.99 (6.69)	73.53 (7.58)	77.85 (8.15)	83.36 (7.93)	< 0.001
ALT, U/L	14.00 (11.00–17.00)	15.00 (12.00–19.00)	17.00 (13.00–23.00)	23.00 (17.00–32.00)	< 0.001
AST, U/L	16.00 (13.00–19.00)	16.00 (13.75-20.00)	17.00 (14.00–21.00)	19.00 (15.00–23.00)	< 0.001
GGT,U/L	12.00 (10.00–15.00)	13.00 (10.00–17.00)	15.00 (12.00–22.00)	21.00 (15.00–30.00)	< 0.001
HDL-C, mmol/L	1.90 (0.35)	1.56 (0.25)	1.33 (0.21)	1.06 (0.19)	< 0.001
Non-HDL-C, mmol/L	2.80 (2.45–3.19)	3.39 (3.00-3.80)	3.86 (3.43–4.31)	4.46 (3.96–4.99)	< 0.001
TC, mmol/L	4.73 (0.76)	4.97 (0.78)	5.22 (0.81)	5.57 (0.88)	< 0.001
LDL-C, mmol/L	2.42 (2.10–2.77)	2.90 (2.56–3.27)	3.27 (2.88–3.68)	3.65 (3.19–4.14)	< 0.001
TG, mmol/L	0.42 (0.32–0.53)	0.60 (0.47–0.75)	0.85 (0.69–1.05)	1.45 (1.13–1.90)	< 0.001
RC, mmol/l	0.38 (0.33–0.43)	0.48 (0.43–0.54)	0.58 (0.53–0.65)	0.78 (0.69–0.92)	< 0.001
FPG, mmol/L	4.97 (0.39)	5.08 (0.40)	5.21 (0.39)	5.34 (0.37)	< 0.001
HbA1c, %	5.14 (0.30)	5.15 (0.31)	5.20 (0.33)	5.23 (0.34)	< 0.001
SBP, mmHg	108.06 (13.08)	111.38 (13.88)	115.86 (14.43)	120.42 (14.83)	< 0.001
DBP, mmHg	66.62 (9.28)	69.27 (9.62)	72.55 (9.93)	76.04 (10.20)	< 0.001
Habit of exercise	671 (18.84%)	639 (17.95%)	634 (17.81%)	524 (14.71%)	< 0.001
Drinking status					< 0.001
Non or small	3087 (86.69%)	3024 (84.94%)	2851 (80.08%)	2840 (79.75%)	
Light	370 (10.39%)	400 (11.24%)	505 (14.19%)	479 (13.45%)	
Moderate	104 (2.92%)	136 (3.82%)	204 (5.73%)	242 (6.80%)	
Smoking status					< 0.001
Non	2845 (79.89%)	2478 (69.61%)	1950 (54.78%)	1469 (41.25%)	
Past	421 (11.82%)	543 (15.25%)	761 (21.38%)	831 (23.34%)	
Current	295 (8.28%)	539 (15.14%)	849 (23.85%)	1261 (35.41%)	
NAFLD	69 (1.94%)	224 (6.29%)	658 (18.48%)	1552 (43.58%)	< 0.001

Values were expressed as mean (standard deviation) or medians (quartile interval) or n (%)

*RC/HDL-C ratio* remnant cholesterol/high-density lipoprotein cholesterol ratio, *BMI* body mass index, *WC* waist circumference, *ALT* alanine aminotransferase, *AST* aspartate aminotransferase, *GGT* gamma-glutamyl transferase, *TC* total cholesterol, *LDL-C* low density lipoprotein cholesterol, *TG* triglyceride, *FPG* fasting plasma glucose, *HbA1c* hemoglobin A1c, *SBP* systolic blood pressure, *DBP* diastolic blood pressure, *NAFLD* non-alcoholic fatty liver disease

### Association of RC/HDL-C ratio with risk of NAFLD

Four multivariate logistic regression models were developed to assess the association between RC/HDL-C ratio and NAFLD risk (Table 2). The unadjusted OR for the association between the RC/HDL-C ratio and NAFLD risk was 2.90 (95% CI 2.76–3.05). After adjusting for age and sex, the RC/HDL-C ratio was associated with an increased risk of NAFLD (Model I: OR 2.46, 95% CI 2.34–2.60). Model II adjusted the non-collinear variables that have an impact of more than 10% on the risk of NAFLD related to the ratio of RC/HDL-C, and the ratio of RC/HDL-C maintained a positive correlation with the risk of NAFLD disease (OR 1.58, 95% CI 1.49–1.67). Model III’s additional adjustment to significant variables in the univariate analysis only slightly weakened the results (OR 1.51, 95% CI 1.43–1.61). In a further fully adjusted model (Model IV), the association between the RC/HDL-C ratio and NAFLD was maintained to the same extent (OR 1.53, 95% CI 1.44–1.63). Additionally, sensitivity analysis showed similar results in the four models, and the risk of NAFLD increased with the increase of RC/HDL-C ratios (*P*-trend < 0.0001).

**Table 2:** Logistic regression analyses for the association between RC/HDL-C ratio and NAFLD in different models

	RC/HDL-C ratio quartile (OR :95% CI)					
	Multivariable analysis (per SD increase)	Q1	Q2	Q3	Q4	*P*-trend
Unadjusted Model	2.90 (2.76, 3.05)	Ref	3.40 (2.58, 4.47)	11.48(8.91,14.78)	39.10 (30.53, 50.07)	< 0.0001
Model I	2.46 (2.34, 2.60)	Ref	2.93 (2.22, 3.86)	8.59 (6.63, 11.12)	25.88 (20.03, 33.44)	< 0.0001
Model II	1.58 (1.49, 1.67)	Ref	1.89 (1.41, 2.53)	3.58 (2.72, 4.73)	6.20 (4.71, 8.18)	< 0.0001
Model III	1.51 (1.43, 1.61)	Ref	1.82 (1.35, 2.45)	3.25 (2.45, 4.32)	5.42 (4.06, 7.22)	< 0.0001
Model IV	1.53 (1.44, 1.63)	Ref	1.80 (1.33, 2.42)	3.25 (2.45, 4.32)	5.49 (4.11, 7.33)	< 0.0001

* RC/HDL-C ratio* remnant cholesterol/high-density lipoprotein cholesterol ratio, *OR* odds ratios, *CI* confidence interval, *SD* standard deviation, *NAFLD* non-alcoholic fatty liver disease

Model I adjusted for sex and age

Model II adjusted for sex, BMI, TC, FPG and SBP

Model III adjusted for sex, age, BMI, ALT, AST, height, habit of exercise, GGT, TC, FPG, HbA1c and SBP

Model IV adjusted for sex, age, BMI, ALT, AST, height, habit of exercise, GGT, TC, FPG, HbA1c, SBP, drinking status and smoking status

### Subgroup analysis of the relationship between RC/HDL-C ratio and the risk of NAFLD

This study investigated the relationship between the RC/HDL-C ratio and the risk of NAFLD in subgroups of different sexes, ages, BMI and habits of exercise. As shown in Table 3, although stratified analysis provided some reference information in different subgroups, we only observed an interaction between sex and RC/HDL-C ratio in relation to NAFLD risk. Among them, when the RC/HDL-C ratio was higher, the risk of NAFLD associated with women was significantly higher [OR: 2.07 (women) vs. 1.50 (men), *P*-interaction < 0.0001)].

**Table 3:** Stratified associations between RC/HDL-C ratio and NAFLD by age, sex, BMI and habit of exercise

Subgroup	unadjusted OR (95%CI) (Per SD increase)	adjusted 0R (95%CI) (Per SD increase)	*P-*interaction
Age (years)			0.0345
18–29	10.71 (5.13, 22.32)	0.73 (1.75, 12.75)	
30–44	3.52 (3.26, 3.79)	1.62 (1.49, 1.76)	
44–59	2.41 (2.24, 2.59)	1.50 (1.38, 1.63)	
≥ 60	1.75 (1.45, 2.12)	1.40 (1.14, 1.72)	
Sex			< 0.0001
Women	4.08 (3.60, 4.63)	2.07 (1.80, 2.38)	
Men	2.21 (2.09, 2.34)	1.50 (1.41, 1.60)	
BMI (kg/m^2^)			0.1560
< 18.5	2.72 (0.55, 13.48)	1.62 (0.23, 11.32	
≥ 18.5, < 25	2.38 (2.23, 2.53)	1.77 (1.66, 1.90)	
≥ 25, < 30	1.90 (1.72, 2.09)	1.54 (1.39, 1.71)	
≥ 30	2.13 (1.39, 3.26)	1.52 (0.98, 2.37)	
Habit of exercise			0.1817
Yes	2.51 (2.22, 2.83)	1.46 (1.29, 1.66)	
No	2.98 (2.82, 3.15)	1.46 (1.29, 1.66)	

* RC/HDL-C ratio* remnant cholesterol/high-density lipoprotein cholesterol ratio, *OR* odds ratios, *CI* confidence interval, *SD* standard deviation, *BMI* body mass index, *NAFLD* non-alcoholic fatty liver disease; other abbreviations as in Table ​1

Adjusted for sex, BMI, TC, FPG and SBP

### Accuracy of RC/HDL-C ratio in identifying NAFLD

**Fig. 1:**
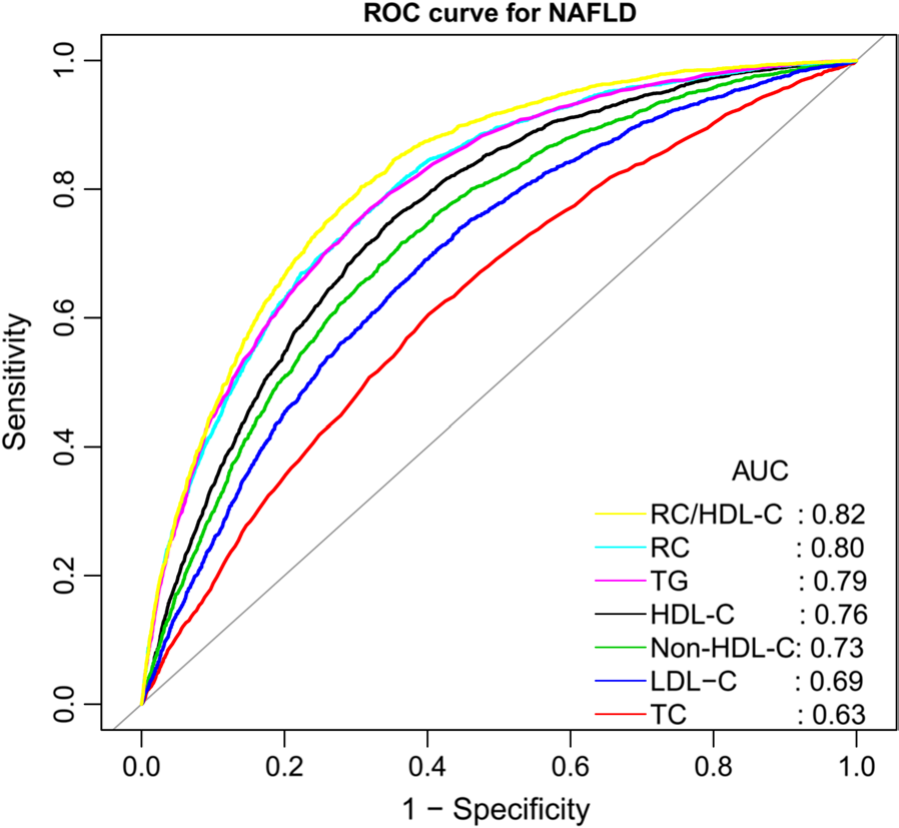
ROC curve analysis of NAFLD-related lipid parameters. ROC: receiver operating characteristic; AUC: area under the curve; HDL-C: high-density lipoprotein cholesterol; TC: total cholesterol; TG: triglyceride; non-HDL-C: non-high-density lipoprotein cholesterol; LDL-C: low-density lipoprotein cholesterol; RC: remnant cholesterol; RC/HDL-C ratio: remnant cholesterol/ high-density lipoprotein cholesterol ratio; NAFLD: non-alcoholic fatty liver disease

ROC curves were used to assess the ability of the RC/HDL-C ratio and other lipid parameters to identify NAFLD (Table 4). As shown in Fig. 1, most of the lipid parameters have moderate discrimination ability for NAFLD. Compared with HDL-C, TC, TG, non-HDL-C, LDL-C and RC, RC/HDL-C ratio has a significantly higher AUC value in the identification of NAFLD (AUC:0.82, all *P* < 0.0001).

**Table 4:** Areas under the receiver operating characteristic curves for each lipid parameters in identifying non-alcoholic fatty liver disease

	AUC	95%CI low	95%CI upp	Best threshold	Specificity	Sensitivity
HDL-C, mmol/L*	0.76	0.75	0.77	1.34	0.65	0.75
TC, mmol/L*	0.63	0.62	0.64	5.21	0.60	0.61
Non-HDL-C, mmol/L*	0.73	0.72	0.74	3.77	0.64	0.72
LDL-C, mmol/L*	0.69	0.68	0.71	3.05	0.56	0.74
RC, mmol/L*	0.80	0.79	0.81	0.58	0.69	0.76
TG, mmol/L*	0.79	0.78	0.80	0.84	0.68	0.77
RC/HDL-C ratio	0.82	0.81	0.83	0.43	0.69	0.80

*AUC* area under the curve, *CI* confidence interval; other abbreviations as in Table ​1

*P < 0.0001, compare with RC/HDL-C ratio by Delong test

## Discussion

In this secondary analysis of the NAGALA cohort, we found that the higher RC/HDL-C ratio in the general population may be closely related to the increased risk of NAFLD. Additionally, our results show that the combination of atherosclerotic lipid HDL-C and RC improves the recognition ability of NAFLD (AUC:0.82), and is significantly better than the traditional lipid parameters. As far as we know, this study proves for the first time that there is a correlation between RC/HDL-C ratio and NAFLD. More importantly, the calculation of the RC/HDL-C ratio is very simple, and this simple new parameter may provide an effective monitoring means of preventing and managing the NAFLD risk in the general population.

Atherogenic dyslipidemia is an important feature of NAFLD and has been recognized as a risk factor for NAFLD [9, 11]. RC is an unconventional lipid, calculated as non-HDL-C – LDL-C [28]. In previous studies, RC has been shown to be a major lipid parameter mediating residual risk of cardiovascular disease, mainly due to its strong atherogenic effect [14–16]. It is reported that RC can promote the formation of foam cells and cause atherosclerosis in many ways. In addition, RC is also involved in the inflammation of the arterial wall, resulting in vascular injury [33–35]. Aside from being closely related to cardiovascular disease, several recent studies have found that higher levels of RC also significantly increase the risk of diabetic complications, hypertension and NAFLD [17–20]. These results suggested that RC has the potential to be used as a predictor of metabolic diseases. HDL-C is an anti-atherosclerotic lipid parameter, and many studies in the past have shown that HDL-C is closely related to a variety of metabolic diseases [9, 10, 36]. So can the combination of RC and HDL-C improve the discrimination ability of NAFLD? Can the combined parameters be used to assess NAFLD risk? In order to solve these problems, a series of analyses were carried out in this study. The study showed that the combination of RC and HDL-C significantly improved the ability of NAFLD identification, and was significantly better than other conventional lipid parameters. Additionally, this study revealed for the first time that RC/HDL-C ratio is an independent risk factor for NAFLD. At present, there are very few studies on RC/HDL-C ratio, and only a few studies have carried out some correlation analysis [21, 22, 37]. As early as 1998, Masuoka et al. described RC/HDL-C ratio for the first time. They evaluated 124 patients who had received coronary angiography, and found that RC/HDL-C ratio was valuable as a predictor of coronary artery disease [37]. Then, several recent studies specifically evaluated the value of the RC/HDL-C ratio in predicting intracranial atherosclerotic and diabetic complications [21, 22]. Combined with our current research, these results suggest that RC/HDL-C ratio is a metabolic-related marker with good potential, which should be paid attention to by more researchers.

In this study, there were some special findings in the subgroup analysis. Subgroup analysis showed that there was a significant interaction between sex and the risk of NAFLD related to the RC/HDL-C ratio, and when the RC/HDL-C ratio was higher, the risk of NAFLD in men was significantly lower than that in women. However, according to the comparison of baseline information in Table 1, it can be seen that the proportion of men was about 5 times higher than that of women in the higher RC/HDL-C ratio group. It may seem odd that there were fewer women in the group with the higher RC/HDL-C ratio but the risk of developing NAFLD is higher. To clarify this particular association, we summarized the baseline characteristics of NAFLD patients according to sex in the highest RC/HDL-C ratio (Q4). As shown in Table 5, women with NAFLD were significantly older than men in Q4, and dyslipidemia appeared more severe in women with NAFLD than in men. Older age in women means aging of the ovaries and decreased estrogen secretion, which leads to a higher risk of NAFLD [38, 39]. In addition, estrogen deficiency in women promotes atherosclerotic lipid abnormalities, visceral weight gain and insulin resistance (IR), which increases the risk of liver disease and heart metabolism [40, 41].

**Table 5:** Baseline characteristics of NAFLD patients in Q4 group of RC/HDL-C ratio

	Women	Men	*P*-value
No. of patients	184	1368	
Age, years	50.00 (42.00–54.00)	43.00 (38.00–50.00)	< 0.001
Weight, kg	64.74 (10.07)	75.47 (10.46)	< 0.001
Height, cm	156.77 (5.03)	170.64 (5.93)	< 0.001
BMI, kg/m^2^	26.31 (3.60)	25.87 (2.95)	0.070
WC, cm	84.79 (9.21)	87.54 (7.10)	< 0.001
ALT, U/L	21.50 (16.00–29.00)	31.00 (23.00–44.00)	< 0.001
AST, U/L	19.00 (16.00–22.00)	21.50 (17.00–27.00)	< 0.001
GGT, U/L	16.00 (13.00–21.00)	26.00 (19.00–37.00)	< 0.001
HDL-C, mmol/L	1.13 (0.18)	1.03 (0.18)	< 0.001
Non-HDL-C, mmol/L	4.73 (4.21–5.30)	4.52 (4.04–5.03)	< 0.001
TC, mmol/L	5.91 (0.88)	5.59 (0.83)	< 0.001
LDL-C, mmol/L	3.85 (3.47–4.35)	3.67 (3.20–4.13)	< 0.001
TG, mmol/L	1.48 (1.21–1.92)	1.60 (1.25–2.16)	0.043
RC, mmol/L	0.81 (0.74–0.95)	0.83 (0.71–0.98)	0.725
RC/HDL-C ratio	0.70 (0.61–0.86)	0.78 (0.65-1.00)	< 0.001
FPG, mmol/L	5.35 (0.37)	5.43 (0.35)	0.003
HbA1c, %	5.46 (0.32)	5.28 (0.34)	< 0.001
SBP, mmHg	124.58 (18.16)	124.67 (14.42)	0.937
DBP, mmHg	77.37 (11.08)	79.08 (10.01)	0.032
Habit of exercise	24 (13.04%)	185 (13.52%)	0.858
Drinking status			< 0.001
Non or small	179 (97.28%)	1110 (81.14%)	
Light	5 (2.72%)	177 (12.94%)	
Moderate	0 (0.00%)	81 (5.92%)	
Smoking status			< 0.001
Non	157 (85.33%)	485 (35.45%)	
Past	9 (4.89%)	391 (28.58%)	
Current	18 (9.78%)	492 (35.96%)	

Values were expressed as mean (standard deviation) or medians (quartile interval) or n (%)

Abbreviations as in Table ​1

The underlying mechanism of the association between RC/HDL-C ratio and NAFLD is still uncertain, but IR may be involved in the association. Previous studies have found that TG to HDL-C ratio was an effective substitute marker for IR and had good IR prediction performance [42, 43], while RC is TG-rich cholesterol, and previous studies have also found that RC was closely related to IR [44]. Therefore, we speculated that RC/HDL-C ratio may be closely related to IR. However, IR information was not measured in this study and therefore could not be evaluated. Further studies are needed to clarify the correlation between RC/HDL-C ratio and IR.

This observational study has several strengths: (1) This study provides the first evidence that RC/HDL-C ratio is independently positively correlated with NAFLD. (2) In this study, a relatively strict statistical model adjustment strategy is implemented, and the sample size is large, so the conclusion of the study can be considered to be reliable. (3) The participants in this study are all people who have undergone physical examination, and the results of the study are very suitable for promotion in the general population.

Several limitations are also worth mentioning: (1) As mentioned above, IR was not measured in this study, so the association mechanism between RC/HDL-C ratio and NAFLD needs to be confirmed by further studies. (2) In this study, NAFLD was diagnosed by ultrasound, and some mild hepatic steatosis may be missed compared with liver biopsy. In addition, the current study excluded people who were taking oral drugs at baseline and people with abnormal blood glucose, which may also lead to the underestimation of the prevalence of NAFLD in the current sample and a certain selection bias. But from another point of view, this study found a correlation between RC/HDL-C ratio and NAFLD in the case of low prevalence, which further suggests that there is a strong correlation between them. (3) In this study, there is a lack of dietary information and some measurement parameters (such as neck and chest circumference), which may help to further understand the relationship between the two. (4) Since this study was designed as a cross-sectional investigation type, it could not prove whether there is a causal relationship between the two, and further prospective studies are needed.

## Conclusion

The work of this study found an independent positive correlation between RC/HDL-C ratio and NAFLD for the first time. Compared with traditional lipid parameters, the RC/HDL-C ratio has a better ability to identify NAFLD in the general population. Based on these strong results, the RC/HDL-C ratio may be a simple, reliable and easy to popularize parameter, which can be used to evaluate the risk of NAFLD in the general population and provide new ideas for improving the risk stratification of NAFLD.

## Supplementary Information

**Additional file 1: Table S1.** Collinearity diagnostics steps.

## Data Availability

The datasets that support the conclusions of this article can be found in the Dryad repository, and we confirm that this data set is publicly available in the Dryad database (https://datadryad.org/stash/dataset/doi:10.5061%2Fdryad.8q0p192).

## Abbreviations

NAFLD: Non-alcoholic fatty liver disease
RC: Remnant cholesterol
HDL-C: High-density lipoprotein cholesterol
ROC: Receiver operating characteristic curve
RC/HDL-C ratio: Remnant cholesterol/high-density lipoprotein cholesterol ratio
TG: Triglycerides
NAGALA: NAfld in Gifu Area, Longitudinal Analysis
ALT: Alanine aminotransferase
AST: Aspartate aminotransferase
GGT: Gamma-glutamyl transferase
TC: Total cholesterol
HbA1c: Hemoglobin A1c
FPG: Fasting plasma glucose
LDL-C: Low-density lipoprotein cholesterol
OR: Odds ratio
CI: Confidence interval
AUC: Area under the curve
IR: Insulin resistance
