# Supplementary material for: Remnant cholesterol/high-density lipoprotein cholesterol ratio is a new powerful tool for identifying non-alcoholic fatty liver disease
Source: BMC Gastroenterol. 2022 Mar 24;22:134. doi: 10.1186/s12876-022-02216-x (PMC8953360; doi:10.1186/s12876-022-02216-x)
Supplement: Supplementary file 1 — Additional file 1: Table S1. Collinearity diagnostics steps. [file 12876_2022_2216_MOESM1_ESM.docx]

Table S1: Collinearity diagnostics steps.

|  | VIF | | | | | | | | |
| --- | --- | --- | --- | --- | --- | --- | --- | --- | --- |
|  | Step 1 | Step 2 | Step 3 | Step 4 | Step 5 | Step 6 | Step 7 | Step 8 | Step 9 |
| RC/HDL-C ratio | 13.5 | 13.5 | 13.5 | 13.5 | 13.4 | 5.7 | 5.7 | 5.7 | 1.6 |
| Sex | 3.3 | 3.3 | 3.3 | 3.3 | 3.3 | 3.2 | 3.2 | 3.2 | 3.2 |
| Age | 1.4 | 1.4 | 1.4 | 1.4 | 1.4 | 1.4 | 1.3 | 1.3 | 1.3 |
| Weight | 169.3 | 169.3 | 169.3 | 169.3 | NA | NA | NA | NA | NA |
| Height | 52.1 | 52.1 | 52.1 | 52.1 | 2.9 | 2.9 | 2.4 | 2.4 | 2.4 |
| BMI | 96.3 | 96.3 | 96.3 | 96.3 | 5 | 5 | 1.6 | 1.6 | 1.6 |
| WC | 5.9 | 5.9 | 5.9 | 5.9 | 5.9 | 5.9 | NA | NA | NA |
| ALT | 4.1 | 4.1 | 4.1 | 4.1 | 4.1 | 4.1 | 4.1 | 4.1 | 4.1 |
| AST | 3.3 | 3.3 | 3.3 | 3.3 | 3.3 | 3.3 | 3.3 | 3.3 | 3.3 |
| GGT | 1.5 | 1.5 | 1.5 | 1.5 | 1.5 | 1.5 | 1.5 | 1.5 | 1.4 |
| GGT | 1.5 | 1.5 | 1.5 | 1.5 | 1.5 | 1.5 | 1.5 | 1.5 | 1.4 |
| HDL-C | Inf | NA | NA | NA | NA | NA | NA | NA | NA |
| TC | Inf | 15.1 | 15.1 | 15.1 | 15.1 | 1.3 | 1.3 | 1.3 | 1.3 |
| Non-HDL-C | Inf | Inf | NA | NA | NA | NA | NA | NA | NA |
| LDL-C | Inf | Inf | Inf | NA | NA | NA | NA | NA | NA |
| TG | Inf | Inf | Inf | 42.6 | 42.4 | 5.1 | 5.1 | 5.1 | NA |
| RC | Inf | Inf | Inf | 116.3 | 115.6 | NA | NA | NA | NA |
| FPG | 1.5 | 1.5 | 1.5 | 1.5 | 1.5 | 1.5 | 1.5 | 1.5 | 1.5 |
| HbA1c | 1.2 | 1.2 | 1.2 | 1.2 | 1.2 | 1.2 | 1.2 | 1.2 | 1.2 |
| SBP | 5.5 | 5.5 | 5.5 | 5.5 | 5.5 | 5.5 | 5.5 | 1.4 | 1.4 |
| DBP | 5.6 | 5.6 | 5.6 | 5.6 | 5.6 | 5.6 | 5.6 | NA | NA |
| Habit of exercise | 1 | 1 | 1 | 1 | 1 | 1 | 1 | 1 | 1 |
| Drinking status | 1.2 | 1.2 | 1.2 | 1.2 | 1.2 | 1.2 | 1.2 | 1.2 | 1.2 |
| Smoking status | 1.4 | 1.4 | 1.4 | 1.4 | 1.4 | 1.4 | 1.4 | 1.4 | 1.4 |
| SBP | 5.5 | 5.5 | 5.5 | 5.5 | 5.5 | 5.5 | 5.5 | 1.4 | 1.4 |
| DBP | 5.6 | 5.6 | 5.6 | 5.6 | 5.6 | 5.6 | 5.6 | NA | NA |

Abbreviations: Inf: infinity; VIF: Variance inflation factor; Other abbreviations as in Table ​1.

Note: VIF = 1/(1-R^2^).
